# Less Might Be More: Conduction Failure as a Factor Possibly Limiting the Efficacy of Higher Frequencies in rTMS Protocols

**DOI:** 10.3389/fnins.2018.00358

**Published:** 2018-05-28

**Authors:** Islam Halawa, Amir Goldental, Yuichiro Shirota, Ido Kanter, Walter Paulus

**Affiliations:** ^1^Department of Clinical Neurophysiology, University Medical Center Göttingen, Göttingen, Germany; ^2^Department of Physics, Bar-Ilan University, Ramat-Gan, Israel; ^3^Goodman Faculty of Life Sciences, Gonda Interdisciplinary Brain Research Center, Bar-Ilan University, Ramat-Gan, Israel

**Keywords:** HfrTMS, rTMS, ITIs, NRFs, neuronal cultures

## Abstract

**Introduction:** rTMS has been proven effective in the treatment of neuropsychiatric conditions, with class A (definite efficacy) evidence for treatment of depression and pain (Lefaucheur et al., 2014). The efficacy in stimulation protocols is, however, quite heterogeneous. Saturation of neuronal firing by HFrTMS without allowing time for recovery may lead to neuronal response failures (NRFs) that compromise the efficacy of stimulation with higher frequencies.

**Objectives:** To examine the efficacy of different rTMS temporal stimulation patterns focusing on a possible upper stimulation limit related to response failures. Protocol patterns were derived from published clinical studies on therapeutic rTMS for depression and pain. They were compared with conduction failures in cell cultures.

**Methodology:** From 57 papers using protocols rated class A for depression and pain (Lefaucheur et al., 2014) we extracted Inter-train interval (ITI), average frequency, total duration and total number of pulses and plotted them against the percent improvement on the outcome scale. Specifically, we compared 10 Hz trains with ITIs of 8 s (protocol A) and 26 s (protocol B) *in vitro* on cultured cortical neurons.

**Results:** In the *in vitro* experiments, protocol A with 8-s ITIs resulted in more frequent response failures, while practically no response failures occurred with protocol B (26-s intervals). The HFrTMS protocol analysis exhibited no significant effect of ITIs on protocol efficiency.

**Discussion:** In the neuronal culture, longer ITIs appeared to allow the neuronal response to recover. In the available human dataset on both depression and chronic pain, data concerning shorter ITIs is does not allow a significant conclusion.

**Significance:** NRF may interfere with the efficacy of rTMS stimulation protocols when the average stimulation frequency is too high, proposing ITIs as a variable in rTMS protocol efficacy. Clinical trials are necessary to examine effect of shorter ITIs on the clinical outcome in a controlled setting.

## Introduction

Repetitive transcranial magnetic stimulation (rTMS) is a non-invasive therapeutic tool for a variety of neuropsychiatric conditions (Lefaucheur et al., 2014). There are presently 617 ongoing clinical trials registered at https://clinicaltrials.gov/ (accessed 05.03.2018). There is class A evidence of the therapeutic utility of rTMS in the treatment of depression and chronic pain (Lefaucheur et al., 2014) which led the FDA to approve the treatment in the USA and Canada for depression (Lefaucheur et al., 2014).

On the other hand, rTMS used in the treatment of other disorders, such as panic disorders, hallucinations, obsessive-compulsive disorder, schizophrenia, Parkinson's disease, dystonia and stroke has been less promising so far (Lefaucheur et al., 2014). To increase the use of rTMS and its acceptance in the medical community across different medical specialties requires a better understanding of the potential pitfalls of the employed protocols.

As far as stimulation frequency is concerned, there is some consensus on the excitatory effects at higher frequencies and inhibitory effects at lower frequencies (Fitzgerald et al., 2006), although further temporal variants also play a role.

However, the temporal organization of rTMS pulses, including the inter-train interval (ITI) or intra-burst interval, has attracted less attention, despite its seemingly cardinal role in determining rTMS efficacy. The relevance of the ITI has been more intensively studied in so-called “patterned stimulation protocols” such as theta burst (Huang et al., 2005) or quadripulse (Hamada et al., 2008) stimulation. In these paradigms, a smaller number of stimulations was more efficacious than rTMS with a constant inter-stimulation interval. However, the influence of the ITI needs to be better addressed in the conventional stimulation protocols used in the treatment of depression or chronic pain.

Different time ranges play different roles in this context. In TBS it seems that the introduction of an 8-s ITI between ten bursts of three high-frequency pulses at 5 Hz (i.e., in a theta range) was facilitatory, while cTBS without this 8-s ITI was inhibitory (Huang et al., 2005). With QPS the effect on plasticity induction is quite the opposite when intra-burst intervals within the burst of four are crossed over from facilitation when using 5 ms to inhibition when using 50 ms (Hamada et al., 2008). Even for conventional 5 Hz rTMS protocols the introduction of a longer ITI switched the aftereffects from inhibition to excitation (Rothkegel et al., 2010).

Another way to view the issue of ITI is to apply information gained from paired-pulse TMS protocols. For example, repetitive paired-pulse stimulation using 10–15 ms intervals, which can cause facilitation in the intracortical facilitation (ICF) protocol, was more excitatory than 2–3 ms intervals, which would cause inhibition in the short-interval intracortical inhibition (SICI) (Sommer et al., 2001; Shirota et al., 2016). In this way, even though ITIs were initially introduced for practical reasons, such as to avoid coil overheating or for probably mistaken safety considerations to reduce the risk of seizures, it can be a critical parameter that determines the clinical efficacy of rTMS.

The problem is that infinite possibilities exist for manipulating intervals. For example, using 10 ms intervals in the short intra-cortical facilitation (ICF) range, repetitive paired-pulse stimulation was more excitatory than with 2 ms intervals both at 5 and 2 Hz repetition frequency (Sommer et al., 2001).

Other strategies for increasing efficacy are increasing the duration of the stimulation and concomitantly the total number of pulses, e.g., 54,000 stimuli over 3 days (George et al., 2014). But this strategy may fail: when the motor evoked potential is used as the biomarker with theta burst, prolonged, intermittent theta burst stimulation (1,200 pulses instead of 600) is inhibitory, i.e., efficacy not only declined, but the direction of the changes was even reversed when the number of stimulation pulses was doubled (Gamboa et al., 2010). tDCS also showed similar behavior with a reversal of aftereffects when two sessions were applied back-to-back compared to a single session (Monte-Silva et al., 2013).

Also the very simple parameter of rTMS frequency still remains insufficiently investigated. Frequencies higher than 10 Hz have rarely been used in conventional rTMS protocols. One reason for this being the increasing technical difficulties, in particular coil heating with higher frequencies or a possibly increased risk of seizures. The safety guidelines (Rossi et al., 2009) at 110% motor threshold assume a safe train duration of more than 5 s at 10 Hz that decreases to 1.6 s at 15 Hz and 0.84 s at 25 Hz.

### Neuronal response failures: from cell cultures to human brains

**Figure 1:**
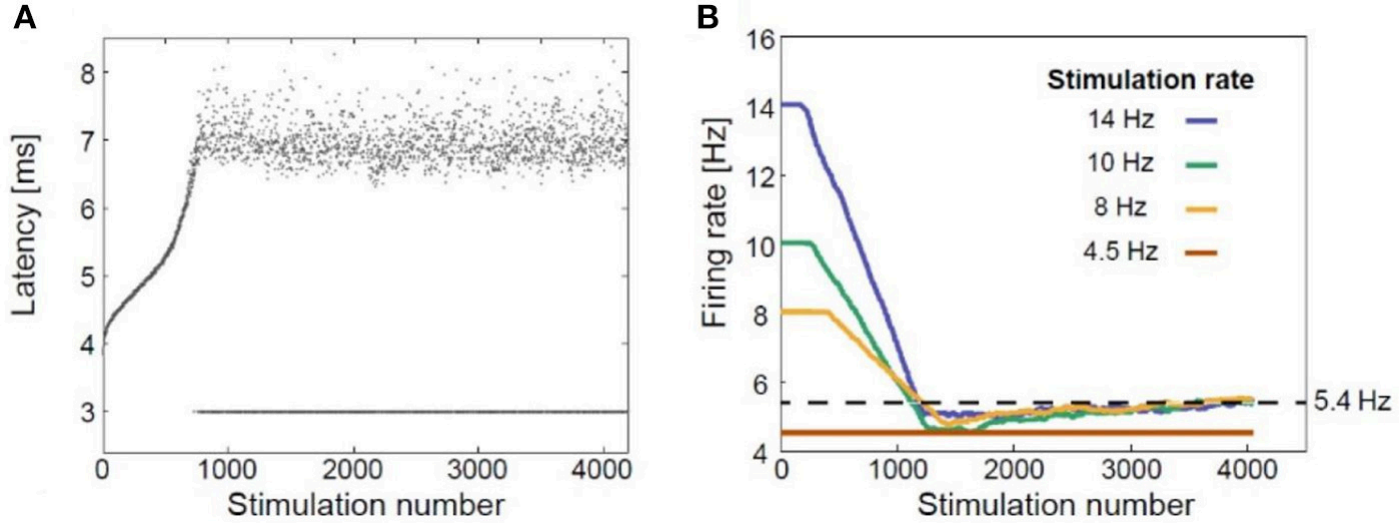
Neuronal plasticity. **(A)** The neuronal response latency (NRL) of a cultured cortical neuron functionally separated from its network using a pharmacologic block, stimulated at a frequency of 10 Hz. Response failures are represented by dots at NRL = 3 ms. **(B)** Firing rates for different stimulation rates using a sliding window of 1,000 stimulations, indicating a saturated firing rate (~5.4 Hz, dashed line) independent of the applied stimulation rate. Adapted from Goldental et al. (2016) with the author's permission.

Given the uncertainty of the significance of the temporal organizations in rTMS, we hypothesized that delayed neuronal response latency (NRL) or a resulting neuronal response failure (NRF) should play an important role in the biological effects of rTMS. When a neuron is subjected to supra-threshold stimulation, it typically produces an action potential, which can be measured extracellularly several milliseconds after the stimulation. The time-gap between the stimulation and the corresponding recorded evoked spike is known as neuronal response latency (Vardi et al., 2012; Goldental, 2014; Sardi et al., 2017, 2018). If the stimulus frequency is low enough, NRL is stable and there are no response failures. With repeated stimulations above a so-called critical frequency (f_c_), the NRL was found to stretch gradually (Figure 1A) (Goldental et al., 2016) until it fluctuated around an average value, and stochastic neuronal response failures (NRFs) appeared, i.e., the average firing rate is saturated and is equal to f_c_, even if the external stimulation frequency is higher. The neuron functions like a low-pass filter. At a stimulation frequency higher than f_c_ NRFs appear randomly and independently with a probability of f_c_/f (Goldental et al., 2015). After several minutes without stimulation, the NRL approaches its initial value (Vardi et al., 2012). On the other hand, when a neuron is stimulated below its f_c_, the NRL is stable (Vardi et al., 2015), and the probability of neuronal response failure is negligible (Vardi et al., 2015).

### Repetitive transcranial magnetic stimulation and neuronal plasticity

Thus, at the single neuron level, the response probability decreases when the stimulation frequency exceeds f_c_ (Figure 1B; Goldental et al., 2016). At the population level, e.g., in recurrent or feed-forward networks, this effect is even enhanced because a sufficient number of neurons must fire in synchrony for the signal to propagate among populations of neurons representing perceptual entities (Vardi et al., 2012).

As a result, on the one hand, we expect rTMS with a lower frequency to be more reliable and thus more efficient than higher frequency stimulation, in which neuronal response lead to less synchronized responses. On the other hand, we must differentiate between intended inhibitory effects that occur with rTMS frequencies around 1 Hz, and rTMS stimulation with frequencies higher than 1 Hz, which are excitatory. The frequency/response curve should therefore resemble an inverted U. The situation is however more complicated when considering intervals, which allow a recovery of conduction reliability as outlined in more detail in the comparison of two trials on rTMS in depression.

The influence of the inter-train interval in rTMS has been investigated less than that of stimulation frequency (e.g., 1 or 5 Hz) even in the context of basic research, so we examined the premise using the neuronal culture developed by Vardi and co-workers (Vardi et al., 2015). The greatest incremental success in the treatment of depression was seen in a key trial (O'Reardon et al., 2007) using a special patterned paradigm based on 10 Hz rTMS, while the results of a different multicenter trial conducted at the same time using 10 Hz rTMS in the treatment of refractory depression were negative (Herwig et al., 2007). Among the many reasons for the contrary outcomes, e.g., total stimulation duration, number of stimuli per session, total duration in days and intensity, the two studies differed in the pattern of their rTMS stimulation sequence. The successful data set (Protocol B) applied 3,000 10 Hz stimuli per day with 4 s of stimulation and an interval of 26 s. The unsuccessful study (Protocol A) applied 2,000 10 Hz stimuli per day with 2 s of stimulation and an 8-s interval without stimulations. It should be noted that the Herwig Protocol (Herwig et al., 2007) used an accelerated treatment protocol of 15 days, while the O'Reardon Protocol (O'Reardon et al., 2007) used a full course of 4–6 weeks of treatment. Treatment duration is also known to be a factor that influences treatment efficacy (Lefaucheur et al., 2014). Nonetheless, ITI duration is also an important parameter consideration, which may influence the efficacy of clinical protocols.

We compared both protocols using a single neuron *in vitro* approach in a cell culture model (Goldental et al., 2016).

In summary, we investigated in a literature review whether conduction failure of cortical neurons limited reliable spike conduction by comparing the published results of clinical studies with *in vitro* experiments.

## Methods

Protocols A and B were compared in a cell culture (Vardi et al., 2012; Sardi et al., 2017). All cell culture experimental procedures were conducted in accordance with the National Institutes of Health Guide for the Care and Use of Laboratory Animals and Bar-Ilan University Guidelines for the Use and Care of Laboratory Animals in Research and are approved and supervised by the Institutional Animal Care and Use Committee. Cortical neurons are obtained from newborn rats within 48 h after birth using mechanical and enzymatic procedures (Vardi et al., 2012; Sardi et al., 2017). The neurons are plated directly onto substrate-integrated multi-electrode arrays (MEAs) and are allowed to develop functionally and structurally mature networks over a period of 2–3 weeks *in-vitro*, prior to the experiments. The number of plated neurons in a typical network is in the order of 1,300,000, covering an area of about 380 mm^2^. In order to conduct experiments in which cultured cortical neurons are functionally isolated from their network, a pharmacological block of glutamatergic and GABAergic synapses is performed. This cocktail does not block the spontaneous network activity completely, but rather makes it sparse. At least 1 h is allowed for stabilization of the effect. For stimulation and recording an array of 60 extracellular electrodes, 30 μm in diameter, and spaced 200–500 μm from each other (Multi-Channel Systems, Reutlingen, Germany) is used. Mono-phasic square voltage pulses were used, in the range of [−800, −500] mV and [60, 400] μs and each channel was sampled at a frequency of 50 k samples/s (Vardi et al., 2015). Post-experiment analyses are performed in a Matlab environment (MathWorks, Natwick, MA, USA).

**Figure 2:**
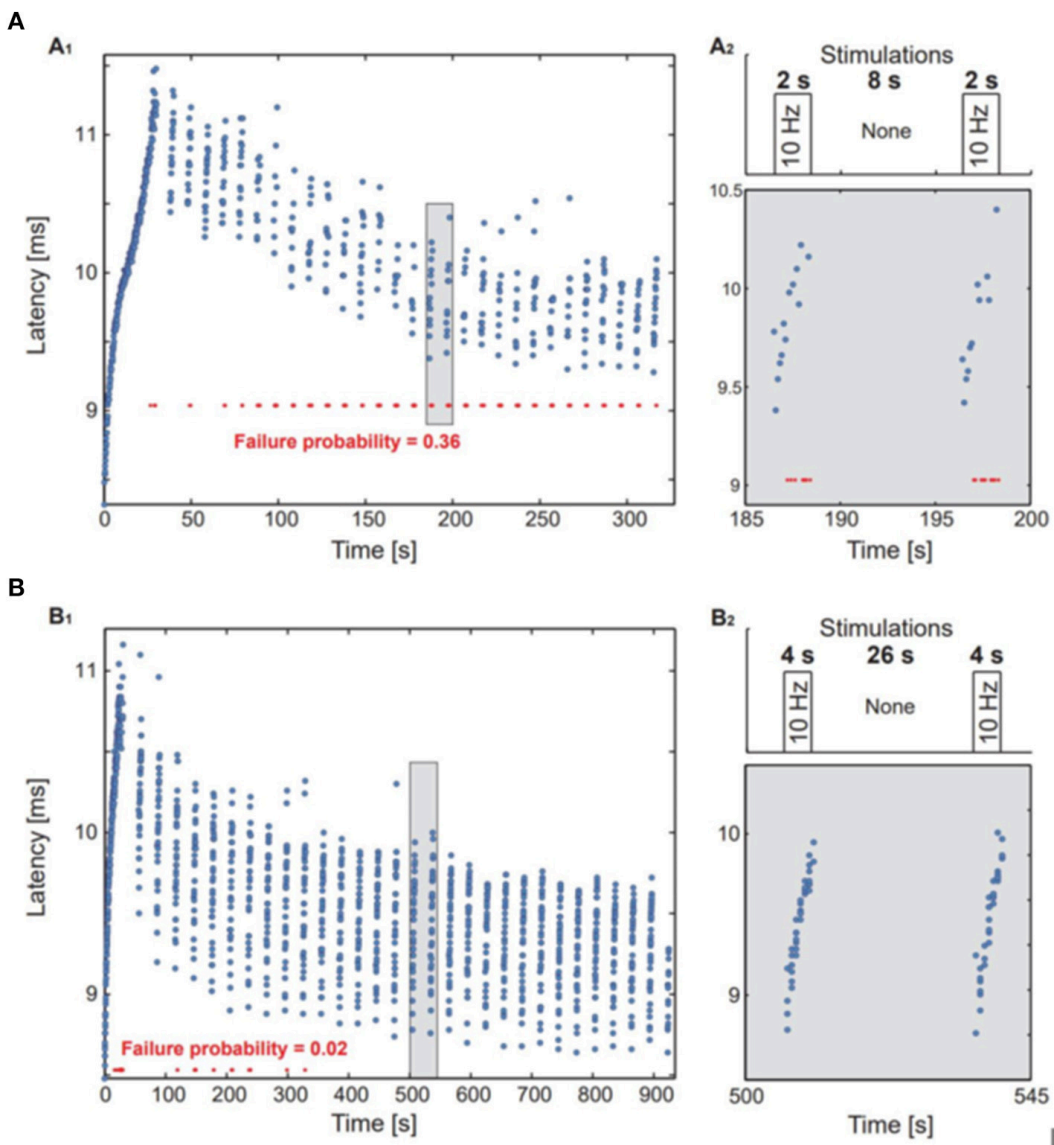
*In vitro* experiments in single neurons. A neuron with an fc = 1.3 Hz was stimulated above the threshold using the stimulation timings of protocols A and B. **(A1,B1)** The neuronal response latency was measured for each response, response failures are represented by red dots. **(A2**,**B2)** upper panel: an illustration of the stimulation protocol. Lower panel: a zoom-in of the marked area at **(A1**,**B1)**.

For a more realistic setting, considering that neurons are not at rest before rTMS, they were primed with 300 stimulations at 10 Hz followed by 30 repetitions of the stimulation patterns according to the protocols A or B (Figure 2).

For the simulation, a critical frequency (f_c_) was randomly chosen from range of 1 to 10 Hz frequency for each neuron. The neuronal response latency was simulated as a constant increase per firing, 0.03 ms, for stimulation frequencies above the neuronal f_c_ and an exponential decay with a time constant of 40 s for lower frequencies. Above the critical neuronal response latency increase of 3 ms neuronal response failures occur and the NRL is set as a random value between 3 and 4 ms for each firing of the neuron. The connections between layers in the feed-forward networks were randomly chosen with a probability of 15/N and a strength equal to 0.1 of the neuronal threshold, where N is the number of neurons in a layer. All other details are the same as in Vardi et al. (2015).

We simulated protocol A neurons in layer 1 with supra-threshold stimulations at 10 Hz for 2 s, every 10 s, and for protocol B we stimulated neurons in layer 1 with supra-threshold stimulations at 10 Hz for 4 s, every 30 s.

To verify the ITI-influence hypothesis from available data on depression studies, we obtained 59 detailed protocols from 57 papers with class A level of evidence. We also focused on the number of pulses per train, number and duration of trains, interval duration, and number of sessions (Tables 1, 2). Forty of the protocols were for depression, with two studies (George et al., 2000; Su et al., 2005) directly comparing two protocols, and 19 were for the treatment of pain as summarized in Lefaucheur et al. (2014). High frequency rTMS stimulation was applied to the left DLPFC for depression, and to the contralateral motor area for pain (Lefaucheur et al., 2014).

**Table 1:** Detailed stimulation patterns and percentage improvement in Hamilton depression scores from all papers using the efficacy A classified HFrTMS protocol in management of depression.

**Study**	**Stimulation frequency /Hz**	**Pulse/burst**	**Burst duration /s**	**Inter train interval/s**	**Repetitions**	**Average frequency /Hz**	**Total duration/s**	**Total number of pulses**	**Intensity RMT**	**Session number**	**Hamilton % improv**	**Hamilton % improv sham**	**Difference**
Pascual-Leone et al., 1996	10	80	8	52	15	1.33	848	1,200	0.9	5	45	7	38
George et al., 1997	20	40	2	60	20	0.65	1,180	800	0.8	10	23	−15	38
Loo et al., 1999	10	50	5	30	30	1.43	1,020	1,500	1.1	10	23	16	7
Padberg et al., 1999	10	50	5	30	5	1.43	145	250	0.9	5	19	1	18
Berman et al., 2000	20	40	2	58	20	0.67	1,142	800	0.8	10	32	3	29
Eschweiler et al., 2000	10	100	10	50	20	1.67	1,150	2,000	1.1	10	20	−6	26
George et al., 2000	5	40	8	22	40	1.33	1,178	1,600	1	10	48	21	27
	20	40	2	28	40	1.33	1,172	1,600	0.9	10	27	21	6
Garcia-Toro et al., 2001a	20	40	2	30	30	1.25	930	1,200	0.9	10	40	9	31
García-Toro et al., 2001b	20	40	2	30	30	1.25	930	1,200	0.8	10	21	15	6
Manes et al., 2001	20	40	2	60	20	0.65	1,180	800	0.9	5	40	28	12
Boutros et al., 2002	20	40	2	58	20	0.67	1,142	800	0.9	10	32	18	14
Padberg et al., 2002	10	100	10	30	15	2.50	570	1,500	1	10	33	13	20
Nahas et al., 2003	5	40	8	22	40	1.33	1,178	1,600	1.1	10	32	25	7
Fregni, 2004	15	75	5	55	40	1.25	2,345	3,000	1.1	10	32	0	32
Hausmann et al., 2004	20	200	10	90	10	2.00	910	2,000	1.1	10	51	40	11
Jorge et al., 2004	10	50	5	60	20	0.77	1,240	1,000	1	10	40	5	35
Koerselman et al., 2004	20	40	2	30	20	1.25	610	800	0.8	10	40	18	22
Mosimann et al., 2004	20	40	2	28	40	1.33	1,172	1,600	1	10	18	16	2
Rossini et al., 2005a	15	30	2	28	20	1.00	572	600	1	10	72	14	58
Rossini et al., 2005b	15	30	2	28	30	1.00	872	900	1	10	52	25	27
Rumi et al., 2005	5	50	10	20	25	1.67	730	1,250	1.2	20	63	30	33
Su et al., 2005	5	40	8	22	40	1.33	1,178	1,600	1	10	54	16	38
	20	40	2	28	40	1.33	1,172	1,600	1	10	58	16	42
Avery et al., 2006	10	50	5	30	32	1.43	1,090	1,600	1.1	15	30	19	11
Herbsman et al., 2009	10	50	5	30	32	1.43	1,090	1,600	1.1	15	32	13	19
Anderson et al., 2007	10	50	5	30	20	1.43	670	1,000	1.1	20	44	15	29
Bortolomasi et al., 2007	20	40	2	28	20	1.33	572	800	0.9	5	52	18	34
Herwig et al., 2007	10	20	2	8	100	2.00	992	2,000	1.1	10	43	38	5
Loo et al., 2007	10	50	5	25	30	1.67	875	1,500	1.1	20	39	26	13
O'Reardon et al., 2007	10	40	4	26	75	1.33	2,224	3,000	1.2	15	25	14	11
Bretlau et al., 2008	8	64	8	52	20	1.07	1,148	1,280	0.9	10	35	23	12
Jorge et al., 2008	10	60	6	60	20	0.91	1,260	1,200	1.1	13	35	15	20
Mogg et al., 2008	10	50	5	55	20	0.83	1,145	1,000	1.1	10	25	10	15
Lisanby et al., 2009	10	40	4	26	75	1.33	2,224	3,000	1.2	20	22	6	16
George et al., 2010	10	40	4	26	75	1.33	2,224	3,000	1.2	20	18	11	7
Paillère Martinot et al., 2010	10	80	8	60	20	1.18	1,300	1,600	0.9	10	42	27	15
Triggs et al., 2010	5	40	8	22	50	1.33	1,478	2,000	1	10	32	22	10
Ray et al., 2011	10	60	6	24	20	2	576	1,200	0.9	10	85	33	52
Baeken et al., 2013, 2014	20	40	2	12	39	2.86	534	1,560	1.1	20	23	18	5

**Table 2:** Detailed stimulation patterns and percentage improvement in visual analog scales from all papers using the efficacy A classified HFrTMS protocol in management of chronic pain.

**Study**	**Stimulation frequency/Hz**	**Pulse/burst**	**Burst duration/s**	**Inter train intervals/s**	**Repetitions**	**Average frequency/Hz**	**Total duration/s**	**Total number**	**Intensity RMT**	**Number of sessions**	**VAS % improv**	**VAS % improv sham**	**Difference**
Lefaucheur et al., 2001a	10	50	5	55	20	0.83	1,145	1,000	0.8	1	20	7	13
Lefaucheur et al., 2001b	10	50	5	55	20	0.83	1,145	1,000	0.8	1	29	−10	39
Lefaucheur, 2004	10	50	5	55	20	0.83	1,145	1,000	0.8	1	35	0	35
Khedr, 2005	20	200	10	50	10	3.33	550	2,000	0.8	5	45	5	40
André-Obadia et al., 2006	20	80	4	84	20	0.91	1,676	1,600	0.9	1	11	8	3
Hirayama et al., 2006	5	50	10	50	10	0.83	550	500	0.9	1	29	5	24
Krishnan and Nestler, 2008	5	500	100	0	1	5.00	100	500	0.95	5	5	10	−5
Lefaucheur et al., 2006	10	60	6	54	20	1.00	1,146	1,200	0.9	1	36	1	35
Saitoh et al., 2007	5	50	10	50	10	0.83	550	500	0.9	1	33	3	30
Saitoh et al., 2007	10	100	10	50	5	1.67	250	500	0.9	1	38	3	35
André-Obadia et al., 2008	20	80	4	84	20	0.91	1,676	1,600	0.9	1	17	2	15
Lefaucheur et al., 2008	10	60	6	54	20	1.00	1,146	1,200	0.9	1	24	9	15
Kang et al., 2009	10	50	5	55	20	0.83	1,145	1,000	0.8	5	23	−3	26
Ahmed et al., 2011	20	200	10	50	10	3.33	550	2,000	0.8	5	54	−2	56
André-Obadia et al., 2011	20	80	4	84	20	0.91	1,676	1,600	0.9	1	12	−2	14
Lefaucheur et al., 2011	10	100	10	30	20	2.50	770	2,000	0.9	1	33	13	20
Hosomi et al., 2013	5	50	10	50	10	0.83	550	500	0.9	1	22	7	15
Jetté et al., 2013	10	50	5	25	40	1.67	1,175	2,000	0.9	1	17	8	9
André-Obadia et al., 2014	20	80	4	84	20	0.91	1,676	1,600	0.9	1	15	3	12

The parameters studied were average stimulation frequency and total duration of stimulation. In the published depression studies, we assessed efficacy as the percentage improvement in the Hamilton depression rating scale (HDRS) compared to sham conditions. In the studies on chronic pain, we assessed changes in pain severity quantified with a visual analog scale (VAS). We plotted the results against average stimulation frequency, interval duration, total duration and total number of pulses. We excluded the study by Fregni (2004) from the analysis because it did not compare rTMS to sham stimulation but only to drug therapy with both showing an equal degree of improvement.

## Results

**Figure 3:**
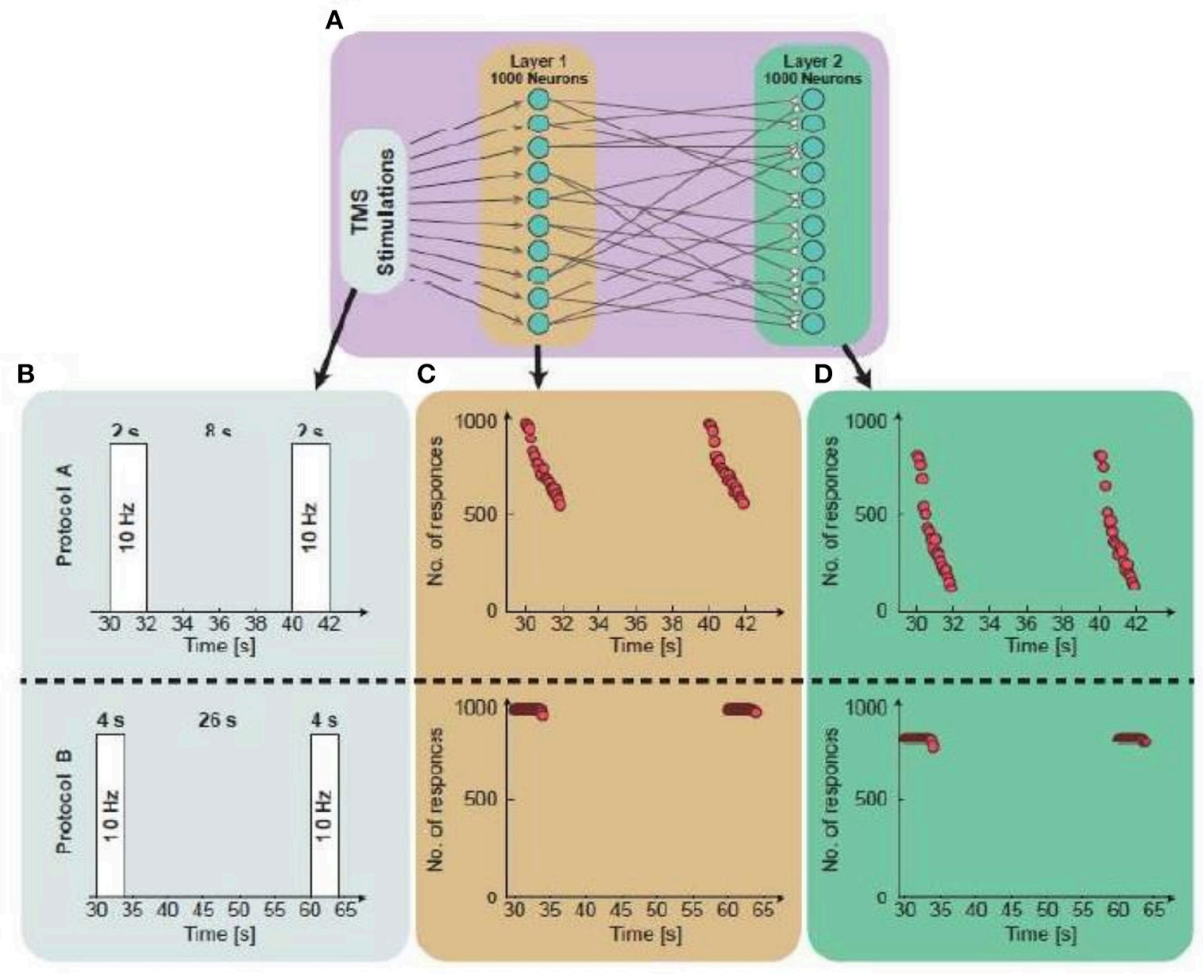
Simulations from a single neuron to population dynamics. **(A)** A scheme of the simulated two-layered feed-forward network: Two populations, 1st layer is randomly connected presynaptically to the 2nd layer. Each layer consists of 1,000 neurons and the 1st layer is stimulated repeatedly. **(B)** An illustration of the simulated protocol. Upper panel: Neurons in the 1st layer are stimulated at 10 Hz for 2 s, every 10 s. Lower panel: Neurons in the 1st layer are stimulated at 10 Hz for 4 s, every 30 s. **(C)** The number of firing neurons in the 1st layer as a function of the stimulation time. The average number of responses in the 1st layer for protocol A and protocol B is 0.75 and 0.999, respectively. **(D)** The number of firing neurons in the 2nd layer as a function of the stimulation time. The response probability in the 2nd layer for protocols A and B is 0.44 and 0.85, respectively.

In the cell culture study, protocol A with the shorter ITIs was associated with a substantial fraction of response failures, while these were much less frequent in protocol B (Figure 3). The average stimulation frequency in protocol A was 2 Hz (20 stimulations per 10 s) as compared to the average stimulation frequency of 1.33 Hz in protocol B (40 stimulations per 30 s).

**Figure 4:**
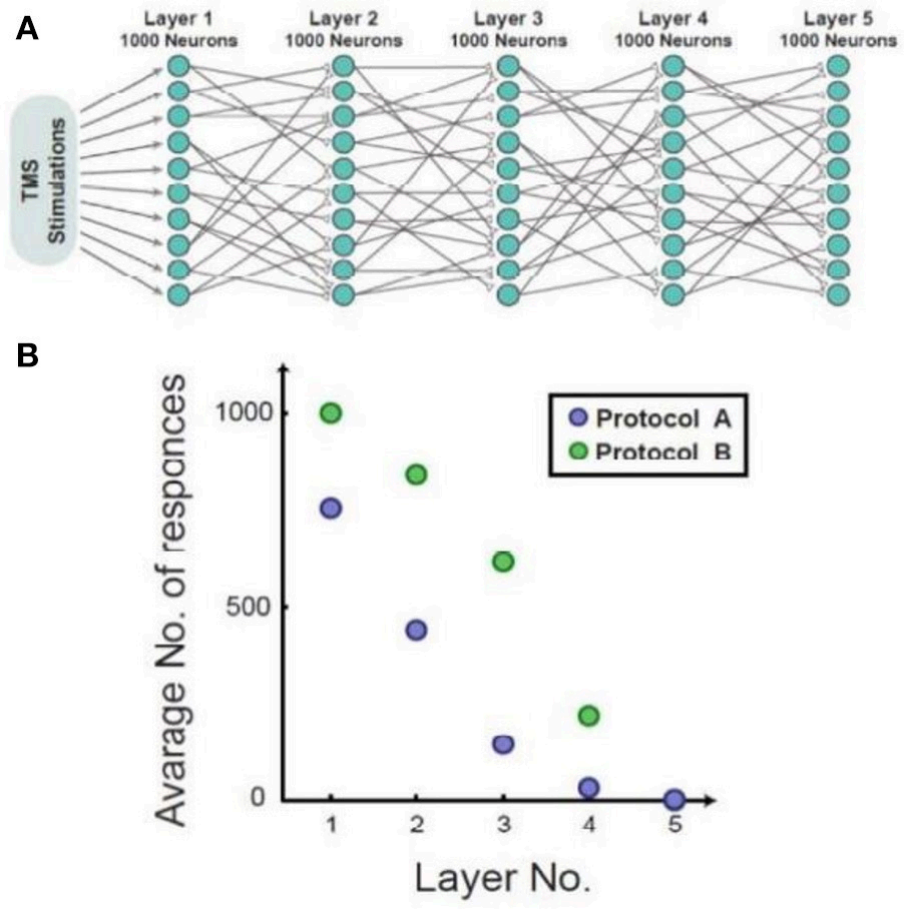
Simulation of a feed-forward network consisting of five layers. **(A)** A diagram of the simulated feed-forward network. **(B)** The average number of responses as a function of the layer number. In the presented case, protocol B demonstrated fewer response failures in comparison to protocol A up to the 5th layer.

Figure 3 illustrates, through simulations, the superiority of protocol B over protocol A for both single neurons and networks, i.e., higher response probability when applying protocol B. Simulations of feedforward, layered networks increasingly demonstrated this effect, which was also visible in simulations including more than two layers (Figure 4).

### HFrTMS protocol evaluation

Our evaluation of the available clinical data in a literature review showed that ITI protocols using ITIs of 20 s and longer were superior to shorter ITI protocols in the treatment of depression and chronic pain. For depression, the relationship between ITIs and improvement in HDRS was not linear and followed an inverted U-curve. We must, however, emphasize that only two studies used ITIs shorter than 20 s in the treatment of depression, and that there was only one such protocol for chronic pain.

We plotted the efficacy against isolated parameters, clustering the data into four groups and connecting the centers of the clusters. The graphs, with the exception of **Figure 6A**, showed low coefficients of determination (*R*^2^), which suggests poor fitting of the graph to the data points. Only the plot of ITIs against VAS improvement in **Figure 6A** showed an *R*^2^ of 0.53 and a *P* of 0.019.

**Figure 5:**
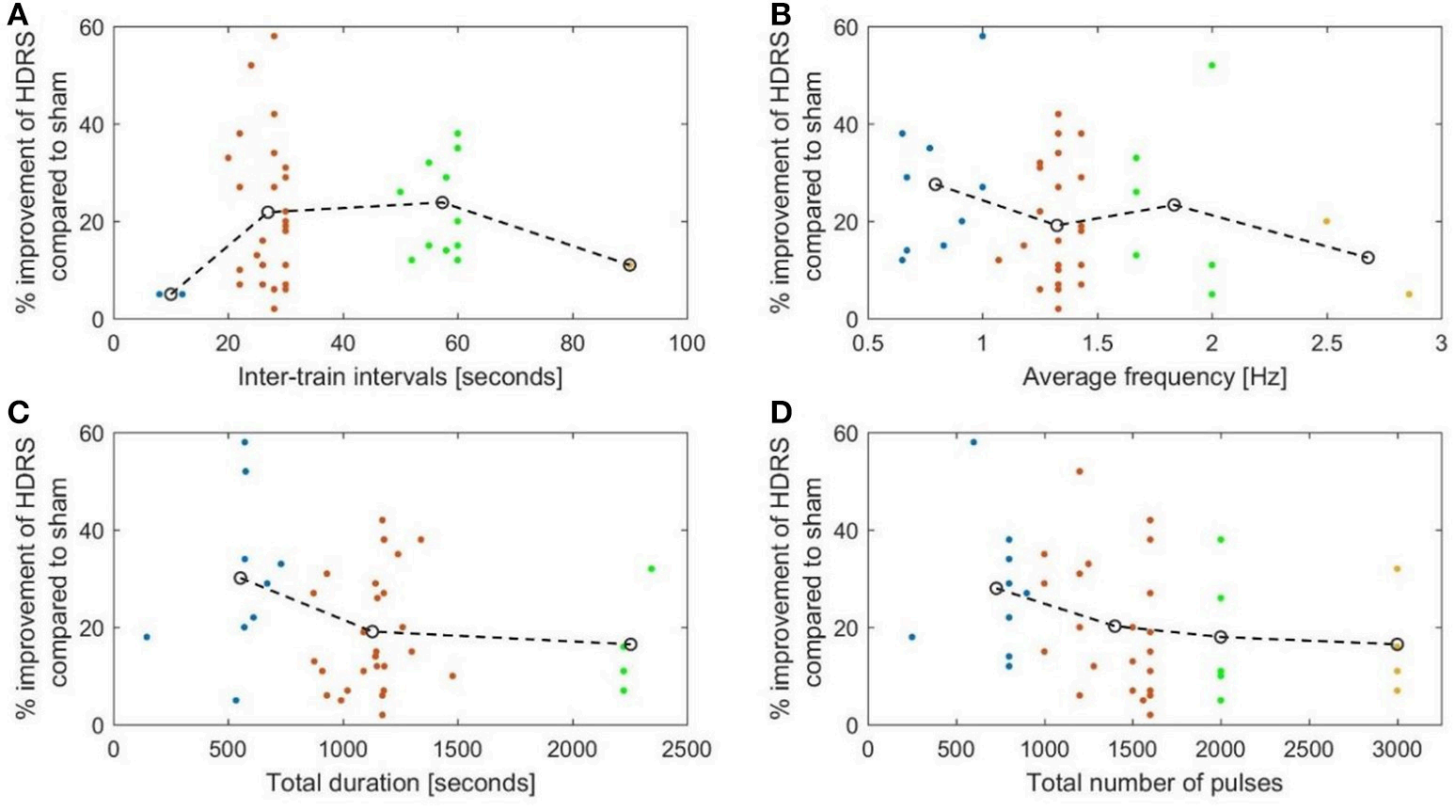
The percentage reduction of the Hamilton depression rating score compared to sham plotted against. **(A)** The duration of the inter-stimulation intervals (*R*^2^ = 0.147). **(B)** The average stimulation frequency (*R*^2^ = 0.143). **(C)** The total duration of stimulation (*R*^2^ = 0.037). **(D)** The total number OF pulses (*R*^2^ = 0.113).

Plotting the inter-train intervals against effect showed that the most effective duration between 10 Hz trains was approximately 50 s (*R*^2^ = 0.147; Figure 5A).

Moreover, the average frequency in the sessions (calculated by dividing the total number of pulses by the total session time in seconds) showed a negative correlation with the efficacy measure, i.e., the higher the average frequency, the less effective the treatment (Figure 5B). As mentioned above, protocol B employed a lower average frequency than protocol A (*R*^2^ = 0.143).

**Figure 6:**
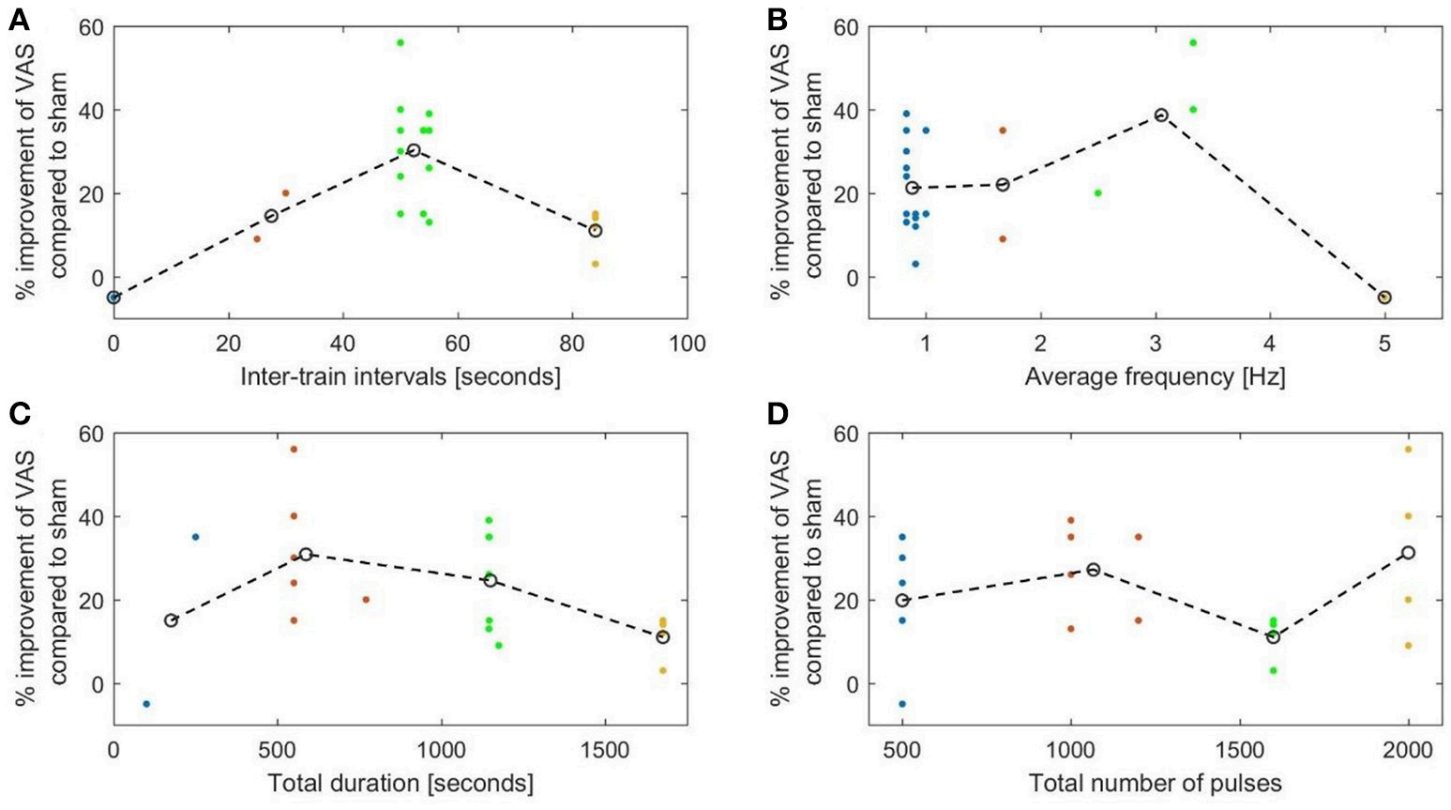
The percentage decrease in VAS rated pain compared to sham plotted against. **(A)** The duration of the inter-stimulation intervals (*R*^2^ = 0.53). **(B)** The length of the average stimulation frequency (*R*^2^ = 0.36). **(C)** The total duration of stimulation (*R*^2^ = 0.33). **(D)** The total number of pulses (*R*^2^ = 0.24).

The evaluated studies on chronic pain revealed similar tendencies (Figures 6A,B) with an optimum inter-train interval also around 50 s (*R*^2^ = 0.53) and an average frequency of about 2.5 Hz (*R*^2^ = 0.36).

Both total duration of stimulation and the number of pulses showed a negative correlation with efficiency in depression studies. Protocols lasting more than 20 min were less effective than shorter ones (Figure 5C) (*P*-value = 0.041). Protocols using more than 2,000 pulses had a lower efficacy than protocols with a smaller number of pulses (Figure 5D) (*P*-value = 0.113 using independent samples *t*-test). Thus, it seems that the average frequency is a more important measure than simply the number of pulses. For example, in our comparison protocol B employed 3,000 pulses lasting approximately 37 min while protocol A, used 2,000 pulses over 16.5 min.

This was also evident in the pain studies (Figure 6C), which showed a decrease in efficiency with longer stimulation (*R*^2^ = 0.33). No correlation was found with the total number of pulses (Figure 6D).

## Discussion

We evaluated the hypothesis that an increase in the effect magnitude of rTMS cannot be achieved by deliberately increasing the number of TMS pulses per time unit because of the provocation of conduction failures. We compared the results of published evidence level A studies in pain and depression with data obtained in cell cultures. In chronic pain protocols we found an inverse relationship between excitatory rTMS frequency and efficacy as predicted, but we were unable to verify this for the depression protocols. We believe that this is because of the differences in the neuronal circuitries involved in the two disorders, with different optimum firing and stimulation frequencies (Krishnan and Nestler, 2008; Simons et al., 2014).

The analysis of the depression studies revealed that most research groups used more or less similar protocols leading to the data being clustered around certain points. Other significant points, e.g., ITIs of ca. 50 s and an average frequency of ca. 1.5 Hz are lacking and should be tested. Despite the fact that relevant points are missing and that the data is noisy, we were still able to detect a pattern in the graphical representations, in which the missing testable variables are easily visible.

In the studies on chronic pain, that of Irlbacher et al. (2006) seems to stand out and illustrates the importance of the ITIs. They used a 100-s continuous train of 5 Hz rTMS (hence the short duration and small number of pulses) without any intervals. Of all the pain studies reviewed here this is the only one that actually showed a worsening of visual analog scales scores compared to sham stimulation. This supports the results of an earlier study that described inhibition using 5 Hz rTMS without intervals (Rothkegel et al., 2010). In all other pain studies the number of pulses per burst ranged from 50 to 200 (for 20 Hz).

We are aware that conduction failures play only one, and probably a minor role compared to the multitude of other plasticity mechanisms. The pleomorphism in response can be mediated by the effect of such patterns and parameters on the behavior of intracellular calcium (Huang et al., 2011; Wilson et al., 2016). Glutamatergic transmission, in particular NMDA receptor mechanisms also play key roles.

A low-pass filter effect of single neurons and, consequently, of neuronal networks is demonstrated by the observation that when a single neuron is stimulated at frequencies of between 20 and 100 Hz, its actual maximum firing frequency is capped at, for example, 17 Hz (Sardi et al., 2016). Even at a continuous 10 Hz stimulation frequency, the neuronal response latencies increase with significant response failures occurring after 700 stimulations.

Neuronal cell culture results showed protocol B to be more efficient than protocol A, with the difference attributed to the longer silence period of 26 s used in protocol B that allowed recovery and relaxation of the NRL toward its initial value. i.e., the higher average stimulation frequency in protocol A is interpreted as a main source of the difference between their efficiencies.

A second finding derived from the cell culture data was that firing was initially stable but that conductions failures occurred after a few hundred stimuli. It is unclear whether this can be transferred to clinical studies, since no similar protocols have been used in patients.

Theoretically, the effect of response failures on the activity of a feed-forward network is enhanced in case of subthreshold synapses. While in the case of a single neuron, the neuron can fire or not-fire, in this case several neurons have to fire synchronously in order to transmit the signal to the next population.

A recent experiment investigated in normal subjects inter-train intervals ranging from 4 to 32 s for a 20 Hz protocol (Cash et al., 2017). At first glance the results seem to argue against our hypothesis; the shortest ITI using 4-s generated the highest MEPs, the 8 s intervals stimulation resulted in the smallest MEPs, which increased again with 16 and 32 s ITI. The 8 to 32 s ITI findings are however in line with our hypothesis. For the 4 s ITI finding a different mechanism may apply: Since SICI was disinhibited most by the shortest ITI of 4 s with a smaller disinhibition at 8 s and almost none at 16 and 32 s. The large 4 s ITI disinhibition of SICI may overrun conduction failures and dominate the 4 s results in the MEP study (Cash et al., 2017). Almost certainly more than one mechanism is involved in the production of the net outcome. Controlled studies are also here necessary to split up the involved mechanisms.

Conduction failures are also determined by the original firing rate of the network so that the upper frequency limit might also apply to inhibitory protocols using 1 Hz stimulation frequencies. Repetition suppression (i.e., the decrease in the amplitude of subsequent MEPs compared to the first stimulus) is the measure to test for inhibitory protocols with lower frequencies (Pitkänen et al., 2017). With higher frequency facilitatory protocols using 5 Hz, the MEP amplitudes increased during the train but inter-train suppression was also detected as a latent period stretching with each TMS pulse (Berardelli et al., 1999).

More data is required in order to optimize the protocol further. In future studies, one might consider starting stimulation with a higher rTMS pulse frequency incorporating a decay over time in order to remain below the critical frequency at which conduction blocks arise. rTMS protocols employing a higher frequency may be more efficient initially but become less reliable over time.

We conclude that depending on the disorder and the desired outcome it should be possible to optimize both the intervals between stimulation trains and the average stimulation frequencies.

In the examined clinical data set for class A rTMS protocols, a lot of heterogeneity were observed in other stimulation variables such as stimulation frequencies, intensities, number of pulses and treatment duration, each one of which might solely explain the discrepancy between the clinical outcome of the O'Reardon et al. (2007) and Herwig et al. (2007) protocols. The focus of our work here was on ITIs, which were so far not considered as an isolated rTMS variable in all studies. Based on the neuronal culture results we propose ITIs as a noteworthy variable in therapeutic rTMS protocols. As such, it would be helpful to initiate specific studies in which only the ITI parameter is modified with a clinical outread.

## Author contributions

IH gathered and analyzed data and wrote Manuscript. AG carried out the neuronal culture experiments, analyzed data and idea. YS gathered and analyzed data and revision. IK oversight and review. WP idea, oversight, and review.

### Conflict of interest statement

The authors declare that the research was conducted in the absence of any commercial or financial relationships that could be construed as a potential conflict of interest.
